# Mutual Life Associations for Physicians and Dentists

**Published:** 1880-10

**Authors:** 


Mutual Life Associations for Physicians and Dentists.—The great, and still increasing popularity of the system of Mutual Life Associations, renders allusion to them in connection with professional men, particularly important just now.

The problem has been plainly solved by those organizations, that life insurance *in them* is not only an *inexpensive*, but perfectly *safe* provision for the families of the assured to the full amount of the policy or certificate, when made in their favor on the death of a member; and equally so, where the amount of insurance is made payable to the member on reaching a certain age, or his becoming permanently disabled by accident or disease.

The reasons stated in the first number of this journal, are equally potent now for the formation of an association of this character, for the benefit of the families of medical men, and should the dentists of Maryland unite with them, such an organization ought to be easily affected; so that an expense of only a few dollars per annum would secure the payment of from three to five thousand dollars to the family of a deceased member, or an equal sum to the member himself on attaining a certain age, or his becoming permanently disabled, and thereby incapable of making support for himself or his family!

The importance of securing provision for those contingencies, seem to us of the greatest magnitude to all, and especially to the classes under consideration. Cannot the professional men of Maryland unite in an association that promises so much to themselves and families? Let some enterprising gentleman in the profession of Baltimore, move in the matter and success will be assured.

